# Use of 3D Models in the Surgical Decision-Making Process in a Case of Double-Outlet Right Ventricle With Multiple Ventricular Septal Defects

**DOI:** 10.3389/fped.2019.00330

**Published:** 2019-08-20

**Authors:** Andrew I. U. Shearn, Michael Yeong, Michael Richard, Maria Victoria Ordoñez, Henry Pinchbeck, Elena G. Milano, Alison Hayes, Massimo Caputo, Giovanni Biglino

**Affiliations:** ^1^Bristol Medical School, University of Bristol, Bristol, United Kingdom; ^2^University Hospitals Bristol, NHS Foundation Trust, Bristol, United Kingdom; ^3^3D LifePrints UK Ltd, Liverpool, United Kingdom; ^4^UCL Institute of Cardiovascular Science, University College London, London, United Kingdom; ^5^Great Ormond Street Hospital for Children, NHS Foundation Trust, London, United Kingdom; ^6^National Heart and Lung Institute, Imperial College London, London, United Kingdom

**Keywords:** double outlet right ventricle (DORV), ventricular septal defect, surgical planning, 3D printing, rapid prototyping, congenital heart defects

## Abstract

3D printing has recently become an affordable means of producing bespoke models and parts. This has now been extended to models produced from medical imaging, such as computed tomography (CT). Here we report the production of a selection of 3D models to compliment the available imaging data for a 12-month-old child with double-outlet right ventricle and two ventricular septal defects. The models were produced to assist with case management and surgical planning. We used both stereolithography and polyjet techniques to produce white rigid and flexible color models, respectively. The models were discussed both at the joint multidisciplinary meeting and between surgeon and cardiologist. From the blood pool model the clinicians were able to determine that the position of the coronary arteries meant an arterial switch operation was unlikely to be feasible. The soft myocardium model allowed the clinicians to assess the VSD anatomy and relationship with the aorta. The models, therefore, were of benefit in the development of the surgical plan. It was felt that the clinical situation was stable enough that an immediate intervention was not required, but the timing of any intervention would be dictated by decreasing oxygen saturation. Subsequently, the oxygen saturation of the patient did decrease and the decision was made to intervene. A further model was created to demonstrate the tricuspid apparatus. An arterial switch was ultimately performed without the LeCompte maneuver, the muscular VSD enlarged and baffled into the neo aortic root and the perimembranous VSD closed. At 1 month follow up SO_2_ was 100%, there was no breathlessness and no echocardiogram changes.

## Background

Over the last 10 years, 3D printing has become an affordable means of quickly producing bespoke parts and models. One application of particular interest is the post-processing of clinical 3D magnetic resonance imaging (MRI) or computed tomography (CT) scans to produce physical models, particularly in the field of congenital heart disease (CHD) (1, 2). This is of considerable interest to clinicians as it can be used to assist in planning surgical procedures or complex interventions, along with having an additional tool to aid in communication between the clinician and the patient/next of kin (3, 4). The technology has now reached the stage where a 3D printing service can be set up in-house in a hospital at a cost that is not prohibitive (5). In order to produce patient-specific CHD models we currently use a desktop 3D printer (Form 2, Formlabs, Somerville, Massachusetts, USA), while also liaising with external partners, such as 3D LifePrints (Liverpool, UK). We report here the use of both rigid and flexible 3D models of a complex CHD case to aid surgical planning. Written, informed consent was obtained from the parents of the patient for the publication of this case report.

## Case Presentation

The patient is a 12-month-old girl with a double outlet right ventricle (DORV), non-committed ventricular septal defects (perimembranous VSD and anterior muscular VSD), aorta (Ao) positioned to the right and posterior to the pulmonary artery (PA) and transposition physiology. The patient was initially palliated with an MPA band to restrict pulmonary blood flow at 16 days of age, followed by an atrial septostomy at 4 months due to low systemic oxygen saturation (SO_2_ 75%). Six months later her case was presented at the medical multi-disciplinary team meeting for planning of further surgical procedures. The two main drawbacks for biventricular repair were the possibly undeveloped left ventricle, and the long distance between the aorta and the VSD, which rendered either a Rastelli or arterial switch procedure more difficult. These conclusions were formed through review of a cardiac MRI. Therefore, a cardiac CT was indicated in order to define the locations of the VSDs and their relationship with the great vessels such that a surgical approach could be established. 3D models were requested to gather extra insight into the cardiovascular anatomy, and particularly to complement the imaging data with regards to the position and size of the VSDs and the position of the coronary arteries. Review of the models enabled a better understanding of the anatomy of the patient and it was felt that a Rastelli was not a viable option due to the long distance between the Ao and the VSD. Therefore, an arterial switch procedure was chosen as a the first option. However, the latter was highly challenging due to the high risk of damaging the chordaes of the tricuspid valve while enlarging the VSD to connect the left ventricle to the PA.

The patient ultimately became more symptomatic for shortness of breath, and the saturation levels dropped from 85% to <75%, secondary to the progression of VSD restriction and an increase in LV pressures to suprasystemic levels. An additional model was made, aiming to emphasize the tricuspid apparatus (leaflets and chordaes) and their proximity with the VSDs. This helped to improve the spatial visualization of these structures, thereby pre-empting potential complications such as tricuspid valve damage or rupture of tricuspid cordes and enabled the planning of the potential strategies needed to decrease the risk of complications.

During the subsequent operation, after opening the pulmonary artery, it was felt that the muscular VSD could be enlarged and, after extensive sub-pulmonary fibrotic and muscular resection, the VSD could be committed to the pulmonary outflow. Furthermore, even though the pre-operative discussion highlighted concerns about the position of the coronary arteries, it was felt that there was a potential for their reimplantation after a more aggressive mobilization. Therefore, an arterial switch operation was performed without the LeCompte maneuver. The muscular VSD was enlarged and baffled into the neo aortic root and extensive muscle resection was performed underneath the pulmonary valve. The very small perimembraneous VSD was closed with a single stich through the tricuspid valve. The postoperative echocardiography showed an excellent repair with no acceleration through both the left and right outflow tracts and the patient was discharged home after 8 days. The patient was followed up 1 month later and showed peripheral SO_2_ of 100%, was asymptomatic for breathlessness and there were no changes on the echocardiogram.

## Three-Dimensional Models

**Figure 1:**
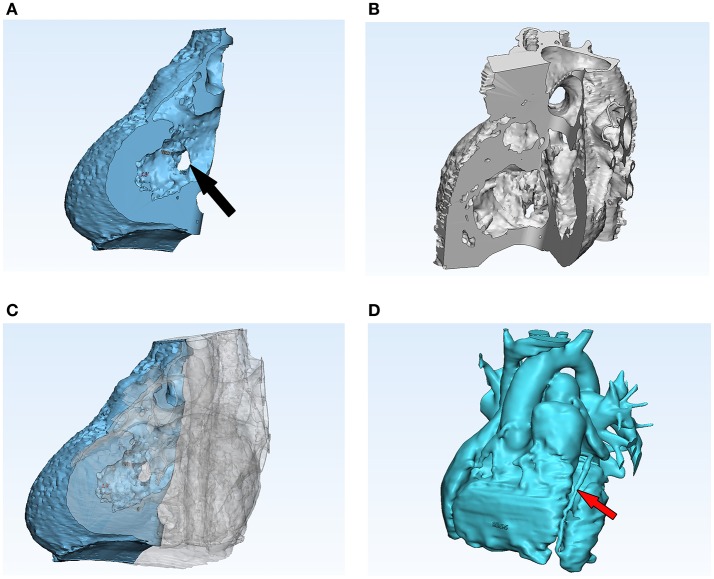
Screenshot of post-processing in 3-Matic. This software was used to cut the myocardium model in such a way that the VSDs could be visualized clearly. The two parts of the model were then printed separately, but fitted together cleanly **(A–C)**. The blood pool model **(D)** was also post-processed in 3-Matic and, while cuts were not required for this model, a 2:1 scale model was produced. The black arrow in **(A)** indicates one of the VSDs and the red arrow in **(D)** a coronary artery.

Under the guidance of an imaging pediatric cardiologist, the blood pool and myocardium were segmented separately, then 3D rendered from the clinically-indicated CT images using commercial software (Mimics v19.0, Materialize, Leuven, Belgium), making use of established segmentation and volume rendering techniques (6). This was then post-processed (3Matic v11.0, Materialize) to clean up and cut the final model, and produce a stereolithography (STL) file which could then be loaded into software for producing the necessary scaffolding specific to the printer (PreForm, Formlabs; Meshmixer, Autodesk, San Rafael, California, USA). During these processing stages, care was taken to emphasize features of particular interest. In consultation with the imaging pediatric cardiologist, a cut was performed virtually such that the final myocardium model would be divided in two parts, the two parts being approximately three quarters and one quarter of the model (Figures 1A–C). This was to facilitate the visualization of the two VSDs. In addition, there was concern over the location of the coronary arteries, so a point was made to segment these and ensure that they could be printed accurately (Figure 1D). In order for the coronary arteries to be visualized easily on the model, the blood pool model was not only printed 1:1 size but also at double its original size, again in consultation with the clinician.

**Figure 2:**
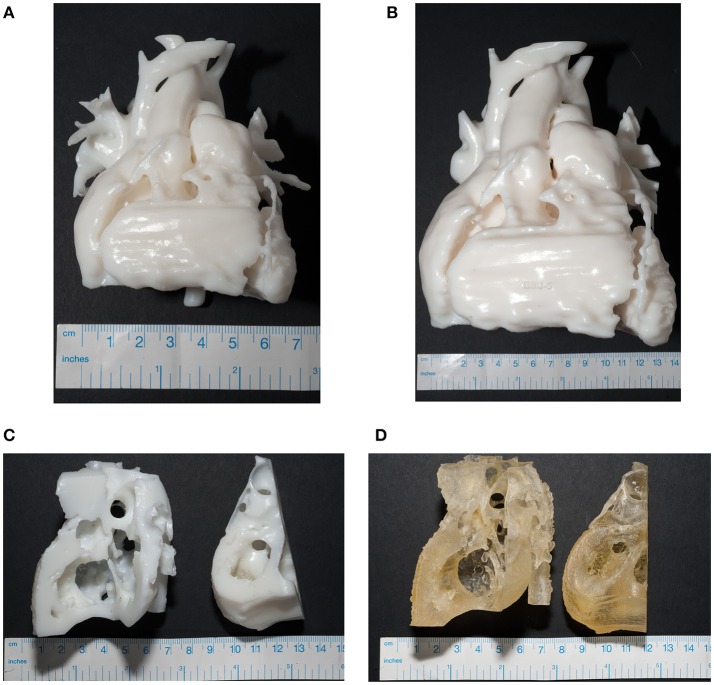
Photographs of the final models, with a measuring tape to demonstrate size. **(A)** The finished blood pool model at 1:1 size. **(B)** The 2:1 sized blood pool model, both produced in Formlabs White resin. Myocardium models were produced in Formlabs White resin **(C)** and using the polyjet printer in Agilus30 **(D)**.

Models of both the blood pool and the myocardium were produced in-house using a Standard White V2 (FLGPWH02) rigid resin (Formlabs). It was felt that also having a model made from a flexible material would allow a more realistic interpretation of the myocardium. Therefore, a myocardium model was also produced in Agilus 30 (Stratasys, Eden Prairie, Minnesota, USA) using an Objet260 Connex 3 printer (Stratasys). Highlighted labels on this model were printed in VeroWhite and VeroMagenta (Stratasys). This is a highly compliant material, with a Shore Hardness of 30–35 Scale A (7). The resulting models made from this scan are shown in Figure 2.

**Figure 3:**
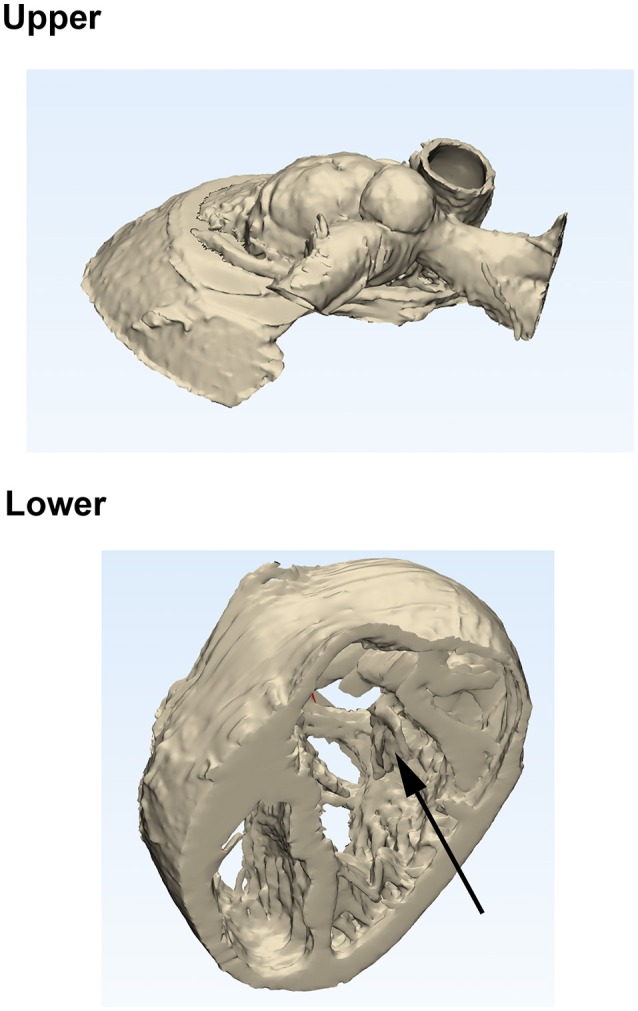
Model produced from later CT scan shortly before surgery. Screenshot from Materialize 3-Matic showing how the model would be cut (**Upper** and **Lower** portions), and demonstrating the tricuspid valve (arrow).

When the patient desaturated and it was decided to intervene, a further CT scan and model were requested. The requirement for this model was to demonstrate the location of the perimembranous VSD, the distance between the aorta and the muscular VSD (for a potential Rastelli procedure), the relationship between the chordae tendineae of the tricuspid valve and the VSD (due to the risk of damaging of the tricuspid valve while repairing the VSD), and the relative location of the great vessels. The model produced was a “hybrid” model, where both hollowed blood pool (to demonstrate the great vessels) and myocardium (to demonstrate the upper portion of the ventricles) were combined into one model. The model was then cut as shown in Figure 3 to facilitate viewing. The model was printed in Formlabs White V4 (FLGPWH04) rigid resin (Formlabs) (not shown).

## Discussion

The models were used to complement information from CT and echocardiography imaging and for assessing the case at the joint multidisciplinary meeting. The surgical review of the initial 3D models suggested that an arterial switch type of operation would be unsuitable due to the unfavorable position of the coronary arteries. The soft 3D model significantly helped in assessing the position of the VSDs regarding the possibility of committing the left ventricle to the aorta and achieving a two-ventricle repair. It was felt that there might be the possibility to enlarge the size of the VSDs by resecting some of the infundibular septum to try and create a channel between the aorta and the left ventricle. The residual right ventricle was observed to be slightly small based on the imaging data, which would possibly have required a Glenn anastomosis at the same time. It was agreed that the patient clinical situation was very stable at the time and there was no strong indication to intervene in the short term, but that a drop in oxygen saturation would mandate a surgical intervention.

The joint discussion between cardiologist and surgeon, using the original group of models, highlighted the fact that the rigid 3D model was very useful for assessing the coronary anatomy and the possibility of coronary transfer for a switch operation. The 3D model also backed up the echocardiographic suggestion of an unfavorable coronary anatomy based on the location of the aorta. Furthermore, it was felt that the soft 3D model helped significantly in assessing the VSD anatomy, their relationship with the aorta and planning the surgical strategy. A further model enabled the visualization and better appreciation of the tricuspid apparatus.

It is interesting to note that, although the assessment of both imaging and models suggested that an arterial switch procedure would not be possible, during the surgery itself it was decided that it was feasible after all. This perhaps demonstrates a limitation of what can be understood from the models (and imaging in general) and that, ultimately, the decision to proceed with the arterial switch came down to the experience of the surgeon in the operating theater. However, this case does demonstrate that the use of 3D models allowed the clinical team to be much better prepared than they otherwise would have been prior to carrying out the surgical intervention.

Overall, 3D printing technology offers the possibility to gather insight into the intra-cardiac structures and relationships between different anatomical components, including baffles and devices (when present), and the size and course of blood vessels, including the coronary arteries. Whilst the latter can also be evaluated using cross-sectional imaging, particularly CT, the advantage of a physical print lies in haptic perception and it is therefore complementary to the visual assessment provided by 3D imaging or other 3D visualization technologies, such as virtual reality and augmented reality.

From a modeling perspective and for the purposes of delineating the anatomy in patients with DORV, it has been recognized that a non-gated MR angiogram is sufficient to identify the ventriculo-arterial relationships for 3D printing. CT imaging, however, should be reserved for patients where additional information on coronary anatomy is needed (8), which was required in the case presented here and, as such, CT imaging was clinically indicated and subsequently used for the 3D model reconstruction. Using 3D technology, personalization of a surgical baffle could also be achieved prior to the surgical procedure (9). Two small case series presenting complex CHD cases (i.e., complex muscular VSD and DORV, DORV and remote VSD) confirmed the role 3D models can play in complex procedures, either confirming or altering the planned strategy, and providing information on the dimensions and orientation of a surgically planned interventricular baffle (10, 11). From a more quantitative standpoint, a study with 25 patients with complex DORV compared operative time and recovery time between a “3D printing group” and a “control group” (i.e., no model) and, although the study sample size was small, it suggests that the insight provided by the model could result in a reduction of cardiopulmonary bypass time, mechanical ventilation time and intensive care unit time (12). Ultimately, these results need to be expanded into larger, randomized, multi-center studies. For instance, a recent prospective case-crossover study involving forty patients with complex CHD showed that the use of a 3D model helped redefine the surgical approach in almost 50% of the cases, including important surgical considerations (e.g., univentricular palliation vs. biventricular repair) (13).

## Concluding Remarks

Given the high complexity of this particular case and the unique anatomy, the input of a 3D model was enormously helpful, not only for the understanding of its particular disease but also for planning a biventricular repair over a univentricular repair and the improvement of the surgical technique. Different models were produced to aid the planning of a complex CHD case, including a rigid model of increased size to appreciate the coronary anatomy, and a soft model cut to provide insight into the position and size of the VSDs.

## Data Availability

No datasets were generated or analyzed for this study.

## Ethics Statement

Written, informed consent was obtained from the legal representatives of the patient.

## Author Contributions

AS, MY, and GB conceived the work. AS, MY, MR, and MO carried out segmentation of images. AS and MR manufactured models. AS wrote the first draft of the manuscript. All authors contributed to the critical revision of the manuscript and approved the submitted version.

### Conflict of Interest Statement

MR and HP are employed by 3D LifePrints. The remaining authors declare that the research was conducted in the absence of any commercial or financial relationships that could be construed as a potential conflict of interest.
